# Microbe-Mineral Interaction and Novel Proteins for Iron Oxide Mineral Reduction in the Hyperthermophilic Crenarchaeon Pyrodictium delaneyi

**DOI:** 10.1128/AEM.02330-20

**Published:** 2021-02-26

**Authors:** Srishti Kashyap, James F. Holden

**Affiliations:** a Department of Microbiology, University of Massachusetts, Amherst, Massachusetts, USA; North Carolina State University

**Keywords:** crenarchaea, cytochromes, denitrification, hydrothermal vents, hyperthermophiles, iron reduction, microbe-mineral interactions, molybdopterin oxidoreductase, proteomics

## Abstract

Dissimilatory iron reduction by hyperthermophilic archaea occurs in many geothermal environments and generally relies on microbe-mineral interactions that transform various iron oxide minerals. In this study, the physiology of dissimilatory iron and nitrate reduction was examined in the hyperthermophilic crenarchaeon type strain Pyrodictium delaneyi Su06. Iron barrier experiments showed that *P. delaneyi* required direct contact with the Fe(III) oxide mineral ferrihydrite for reduction. The separate addition of an exogenous electron shuttle (anthraquinone-2,6-disulfonate), a metal chelator (nitrilotriacetic acid), and 75% spent cell-free supernatant did not stimulate growth with or without the barrier. Protein electrophoresis showed that the *c*-type cytochrome and general protein compositions of *P. delaneyi* changed when grown on ferrihydrite relative to nitrate. Differential proteomic analyses using tandem mass tagged protein fragments and mass spectrometry detected 660 proteins and differential production of 127 proteins. Among these, two putative membrane-bound molybdopterin-dependent oxidoreductase complexes increased in relative abundance 60- to 3,000-fold and 50- to 100-fold in cells grown on iron oxide. A putative 8-heme *c*-type cytochrome was 60-fold more abundant in iron-grown cells and was unique to the *Pyrodictiaceae*. There was also a >14,700-fold increase in a membrane transport protein in iron-grown cells. For flagellin proteins and a putative nitrate reductase, there were no changes in abundance, but a membrane nitric oxide reductase was more abundant on nitrate. These data help to elucidate the mechanisms by which hyperthermophilic crenarchaea generate energy and transfer electrons across the membrane to iron oxide minerals.

**IMPORTANCE** Understanding iron reduction in the hyperthermophilic crenarchaeon Pyrodictium delaneyi provides insight into the diversity of mechanisms used for this process and its potential impact in geothermal environments. The ability of *P. delaneyi* to reduce Fe(III) oxide minerals through direct contact potentially using a novel cytochrome respiratory complex and a membrane-bound molybdopterin respiratory complex sets iron reduction in this organism apart from previously described iron reduction processes. A model is presented where obligatory H_2_ oxidation on the membrane coupled with electron transport and either Fe(III) oxide or nitrate reduction leads to the generation of a proton motive force and energy generation by oxidative phosphorylation. However, *P. delaneyi* cannot fix CO_2_ and relies on organic compounds from its environment for biosynthesis.

## Abstract (precis)

Understanding iron reduction in the hyperthermophilic crenarchaeon Pyrodictium delaneyi provides insight into the diversity of mechanisms used for this process and its potential impact in geothermal environments. The ability of *P. delaneyi* to reduce Fe(III) oxide minerals through direct contact potentially using a novel cytochrome respiratory complex and a membrane-bound molybdopterin respiratory complex sets iron reduction in this organism apart from previously described iron reduction processes.

## INTRODUCTION

Fe(III) oxide minerals are widely available electron acceptors in many hot microbial ecosystems, including the deep hot subsurface (1, 2) and deep-sea hydrothermal vents (3). Dissimilatory Fe(III) oxide mineral reduction is a significant subsurface microbial process occurring across diverse groups of bacteria and archaea (2). It was suggested to be a predominant metabolic process for hyperthermophilic archaea living in iron-rich, circumneutral, mildly reducing hydrothermal environments (4, 5). Yet, our understanding of the growth and ecological constraints, sources of Fe(III) oxide for reduction, types of mineral transformations catalyzed, and physiological mechanisms of reduction in hyperthermophilic archaea is nascent.

Most of what is known about the physiology of dissimilatory iron reduction was determined in model mesophilic bacteria, namely, *Geobacter* and *Shewanella* (see reference 6 for a review). Studies with these organisms showed that Fe(III) oxide mineral reduction involves transfer of electrons from the cytoplasm to the exterior surface of the cell with concomitant generation of a proton gradient across the cytoplasmic membrane followed by transfer of electrons from the cell surface to the mineral. For the first step, multiheme *c*-type cytochromes were critical for electron transfer across the cell wall in both *Geobacter* and *Shewanella* (6). For the second step, the mechanisms described included direct cell contact with minerals; reduction and release of extracellular electron shuttles for nonenzymatic, extracellular iron reduction; and secretion of iron chelators that solubilize iron and return it to the cell for reduction (6). The physiological mechanisms used by mesophilic bacteria to reduce Fe(III) oxide minerals represent general principles for transferring electrons from the cytoplasmic membrane to the mineral. These mechanisms may or may not be the same in hyperthermophilic archaea but represent a starting point for characterizing iron mineral reduction by them. Iron reduction mechanisms and electron transfer proteins in archaea likely differ from those in Gram-negative bacteria because archaea lack an outer cell wall membrane, peptidoglycan, and a periplasm.

Like mesophilic iron reducers, a range of reduction strategies was identified among hyperthermophilic iron-reducing archaea. For example, the crenarchaeon Pyrobaculum islandicum and the euryarchaea Ferroglobus placidus and Geoglobus ahangari required direct mineral contact for reduction and did not appear to secrete extracellular compounds to mediate reduction (7–9). In contrast, Pyrobaculum aerophilum and the thermoacidophilic crenarchaeon *Acidianus* strain DS80 did not require direct mineral contact for Fe(III) oxide mineral reduction (10, 11). Furthermore, unlike mesophilic bacteria, iron reduction in hyperthermophilic archaea was postulated to be dependent on (8, 9) and independent of (7, 10, 11) *c*-type cytochromes, depending on the organism.

In this study, the mechanisms and proteins involved in dissimilatory Fe(III) oxide mineral reduction in the hyperthermophilic H_2_-oxidizing crenarchaeon type strain Pyrodictium delaneyi Su06 were examined. *P. delaneyi* was isolated from a deep-sea hydrothermal vent “black smoker” chimney (4, 5). It grows on a wide range of synthetic nanophase Fe(III) oxide minerals, such as ferrihydrite, lepidocrocite, akaganeite, goethite, hematite, and maghemite (12), but cannot grow on soluble Fe(III) (5, 12). It also uses nitrate as a terminal electron acceptor. The complete genome of *P. delaneyi* Su06^T^ was sequenced (GenBank accession number CP013011), which encodes for 17 predicted *c*-type cytochrome proteins, 11 that are multiheme and 15 that are unique to this family (13). Twelve of the putative *P. delaneyi c*-type cytochrome proteins were predicted to be part of membrane-bound reductase complexes encoded in five putative operons. None of these respiratory complexes have homologs in *Pyrobaculum*, *Geoglobus*, *Ferroglobus*, or *Acidianus*. None of the *P. delaneyi c*-type cytochrome proteins are homologous to the proteins found in *P. islandicum* and *P. aerophilum*. Only two *c*-type cytochrome proteins in *P. delaneyi* showed homology with proteins found in *F. placidus* and *G. ahangari*. Therefore, the *c*-type cytochrome proteins found in *P. delaneyi* were largely unique among iron-reducing archaea.

A combination of dialysis membranes to restrict physical access between *P. delaneyi* cells and minerals and differential proteomic analyses were used to determine if direct mineral contact was needed for reduction of the Fe(III) oxide mineral ferrihydrite and if *c*-type cytochromes or other proteins were differentially produced during growth on iron versus nitrate. Results identified both microbe-mineral interaction and differential protein abundances in hyperthermophilic iron reduction, thus potentially expanding currently known proteins and electron transfer mechanisms for iron mineral reduction. *P. delaneyi* is also typically grown with CO_2_ and trace yeast extract, but it is unclear if the organism can fix CO_2_ using a modified reverse tricarboxylic acid cycle. Therefore, proteomics and enzyme activity assays were used to address this question.

## RESULTS

### Iron barrier experiments.

**FIG 1:**
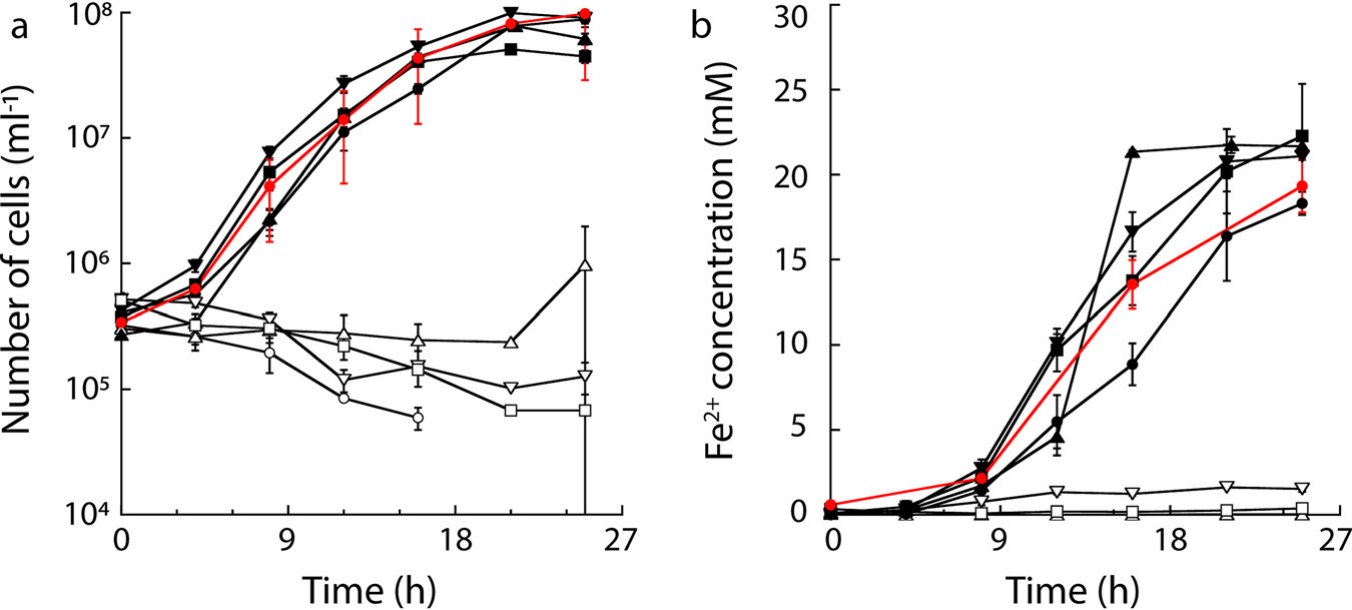
(a) Growth of *P. delaneyi* with (open symbols) and without (filled symbols) a dialysis tubing barrier separating the cells from the ferrihydrite with the addition of 50 μM anthraquinone-2,6-disulfonate (AQDS) as an electron shuttle (▴), 4 mM nitrilotriacetic acid (NTA) as an iron chelator (▾), 75% (vol/vol) cell-free spent supernatant (■), and no addition (●). Cells grown on ferrihydrite in the presence of unfilled dialysis tubing only are shown as a control (red circles). (b) Production of Fe^2+^ when *P. delaneyi* was grown with or without the barrier with the supplements above.

*P. delaneyi* showed hydrogenotrophic growth at 90°C and a concomitant increase in Fe^2+^ production in all experimental conditions where ferrihydrite was available as a free suspension (Fig. 1). CO_2_ in the headspace and 0.02% yeast extract were available as carbon sources. The doubling time for *P. delaneyi* grown on ferrihydrite without a dialysis tube barrier (12- to 14-kDa molecular weight cutoff [MWCO]) was 2.4 h (growth rate ± 95% confidence interval, *k *= 0.29 ± 0.04 h^−1^). Moreover, the doubling times for cells grown on ferrihydrite without a barrier were unaffected by the addition of 50 μM anthraquinone-2,6-disulfonate (AQDS) as an electron shuttle (*k *= 0.32 ± 0.07 h^−1^), 4 mM nitrilotriacetic acid (NTA) as an iron chelator (*k *= 0.26 ± 0.07 h^−1^), or 75% (vol/vol) cell-free spent supernatant (*k *= 0.25 ± 0.07 h^−1^). In contrast to growth without a barrier, *P. delaneyi* was unable to grow with or reduce ferrihydrite sequestered in a dialysis tube barrier (Fig. 1). The separate addition of an exogenous electron shuttle, a chelator, and spent supernatant did not rescue growth or promote iron reduction (Fig. 1). To ensure that the dialysis tubing was not toxic for growth, a control was included where *P. delaneyi* was grown on a free suspension of ferrihydrite in the presence of empty dialysis tubing. In this case, cells showed comparable doubling time (*k *= 0.28 ± 0.04 h^−1^) and iron reduction to all experimental conditions where ferrihydrite was available as a free suspension (Fig. 1). These findings collectively indicated that *P. delaneyi* required direct contact with ferrihydrite for reduction.

### Differential proteomics.

**FIG 2:**
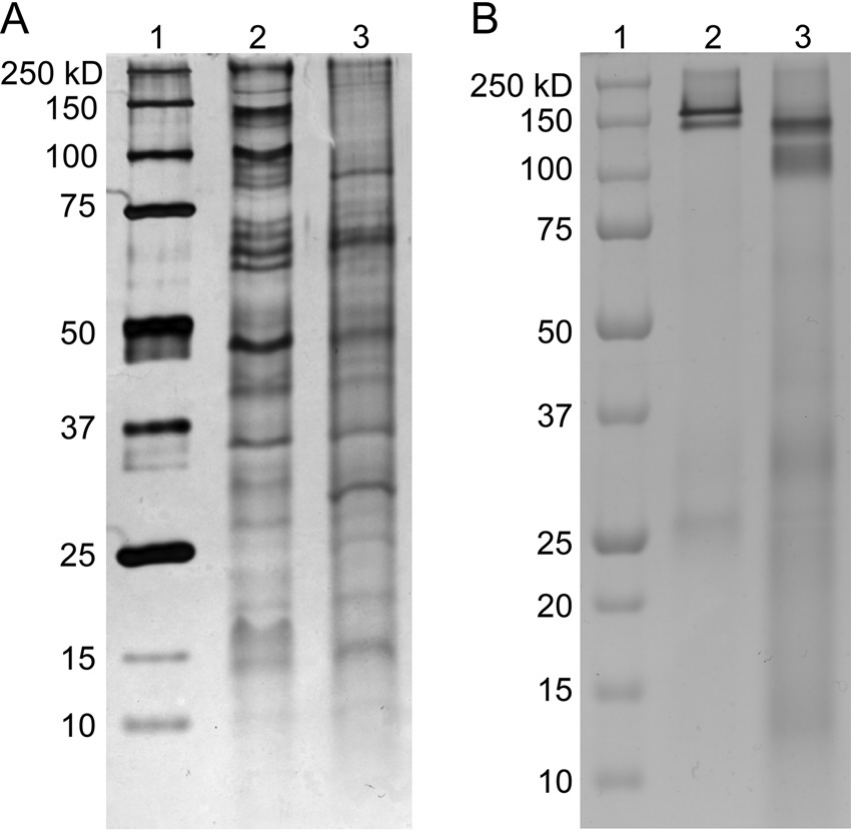
SDS-PAGE (8% tris-tricine) and silver (A) and heme (B) staining of whole-cell proteins extracted from *P. delaneyi* grown at 90°C with either nitrate (lanes 2) or ferrihydrite (lanes 3) as the terminal electron acceptor. Protein standards of known molecular masses are shown in lane 1.

Whole-cell protein extracts separated by sodium dodecyl sulfate-polyacrylamide gel electrophoresis (SDS-PAGE) and visualized separately using silver and cytochrome *c* heme staining showed differential protein and *c*-type cytochrome production when cells were grown on ferrihydrite relative to nitrate (Fig. 2). Protein bands with molecular masses of 140 kDa, 100 kDa, and 63 kDa in the nitrate whole-cell extract were absent in the ferrihydrite whole-cell extract (Fig. 2A). Faint but distinct bands at 85 kDa, 30 kDa, and 25 kDa were visible in ferrihydrite whole-cell extract but nearly absent in nitrate whole-cell extract (Fig. 2A). Heme staining showed distinct differences in *c*-type cytochrome composition between the two growth conditions, with two sharp bands at 145 kDa and 160 kDa in the nitrate whole-cell extract and two broad bands at 140 kDa and 110 kDa in the ferrihydrite whole-cell extract (Fig. 2B). Proteins in the 160-kDa band in the nitrate extract and the 140-kDa band in the ferrihydrite extract were identified using mass spectrometry. These bands were composed of several proteins. An octaheme *c*-type cytochrome (Pyrde_0784) was uniquely identified in the ferrihydrite band. It had one transmembrane spanning sequence indicating that it was a membrane-bound protein. It is part of a putative respiratory complex (Pyrde_0784-Pyrde_0787) that includes a 13-heme *c*-type cytochrome (Pyrde_0786) (see Fig. S1 in the supplemental material), but neither this protein nor associated proteins (Pyrde_0785 and Pyrde_0787) were identified in the heme-excised band. A monoheme *c*-type cytochrome (Pyrde_0283), which is annotated as a putative glutamate synthase, was uniquely identified in the nitrate-excised band. Three *c*-type cytochromes, an octaheme (Pyrde_0496), a monoheme (Pyrde_0921), and a diheme (Pyrde_1256) protein, were identified in both nitrate- and ferrihydrite-excised bands. The octaheme *c*-type cytochrome (Pyrde_0496) is part of a putative membrane respiratory complex containing three other *c*-type cytochromes (Pyrde_0485 [monoheme], Pyrde_0488 [tetraheme], Pyrde_0492 [monoheme]) and two genes related to sulfur reductases (Pyrde_0486 and Pyrde_0487) (Fig. S1). Among these associated proteins, Pyrde_0486 was identified in the nitrate band but not the ferrihydrite band. The other two *c*-type cytochromes identified in both ferrihydrite and nitrate conditions were a 4Fe-4S containing molybdopterin-oxidoreductase protein (Pyrde_0921) and a split-Soret *c*-type cytochrome (Pyrde_1256). The former protein is part of putative membrane molybdopterin oxidoreductase complex (Pyrde_0919-Pyrde_0921) whose function is unknown (Fig. S1). Pyrde_0919 and Pyrde_0920 were also identified among the proteins detected for the excised heme-bands of ferrihydrite and nitrate extracts.

**FIG 3:**
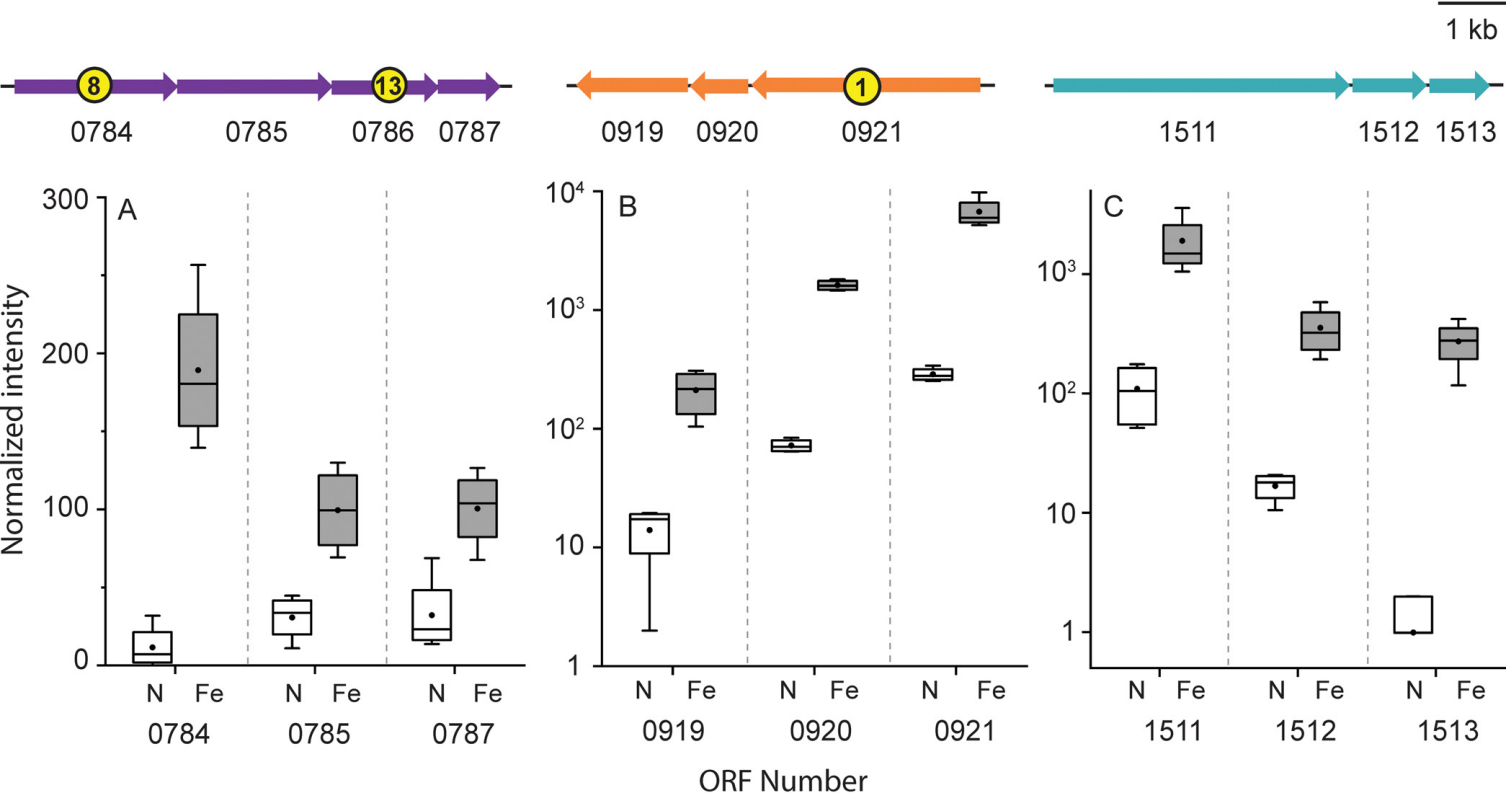
Normalized intensities for proteins in a novel membrane-bound, cytochrome *c*-containing respiratory complex (0784 to 0787) (A) and two novel membrane-bound, molybdopterin-containing respiratory complexes (0919 to 0921, 1511 to 1513) (B, C) for nitrate (N) and ferrihydrite (Fe) grown cells shown in open versus gray shading, respectively. Proteins are described by their open reading frame (ORF) number or Ensembl ID and are shown as part of individual operons (drawn to scale). The yellow circles indicate the number of CXXCH heme motifs in each protein. Each box represents the interquartile range, with the top and bottom of the box depicting the first quartile and third quartile, respectively. The vertical bar depicts the median, the dot depicts the mean, and error bars depict the minimum and maximum intensities.

While the presence of several proteins was confirmed using mass spectrometry of excised heme-stained bands, protein abundance could not be determined with confidence using this method. To accomplish this, peptides were tandem mass tagged (TMT), and differential proteomic analysis was performed using tandem mass spectrometry. Of the 2,037 putative proteins encoded in the *P. delaneyi* genome, 939 proteins were detected by mass spectrometry (see Tables S1 to S4 in the supplemental material). A total of 660 of these proteins were tagged with isobaric labels and used for differential analysis (Tables S1 to S3). Eight protein samples across two growth conditions were examined using principal-component analysis (PCA) (see Fig. S2A in the supplemental material) and correlation analysis between each of the samples (Fig. S2B). Differential proteomic analysis showed differential production of 127 proteins, of which 64 and 63 proteins showed more than 2-fold greater and 2-fold lower abundance, respectively, for ferrihydrite relative to nitrate growth (Tables S1 and S2). Among these proteins was the putative 8-heme *c*-type cytochrome observed in the heme stain gel above (Pyrde_0784) that was 60-fold more abundant in ferrihydrite-grown cells (Fig. 3A; see also Table S1). This cytochrome is part of a putative membrane-bound respiratory complex (Pyrde_0784-Pyrde_0787) containing another 13-heme *c*-type cytochrome that was detected in the mass spectrometry data but not tagged (Table S4). The other two proteins (Pyrde_0785 and Pyrde_0787) were not differentially produced (at the adjusted *P *< 0.01 level) (Fig. 3A; see also Table S3). Two putative membrane-bound molybdopterin-dependent oxidoreductase complexes (Pyrde_0919-Pyrde_0921 and Pyrde_1511-Pyrde_1513) also increased in relative abundance 60- to 3,000-fold and 50- to 100-fold, respectively, in cells grown on ferrihydrite (Fig. 3B and C; see also Table S1).

**FIG 4:**
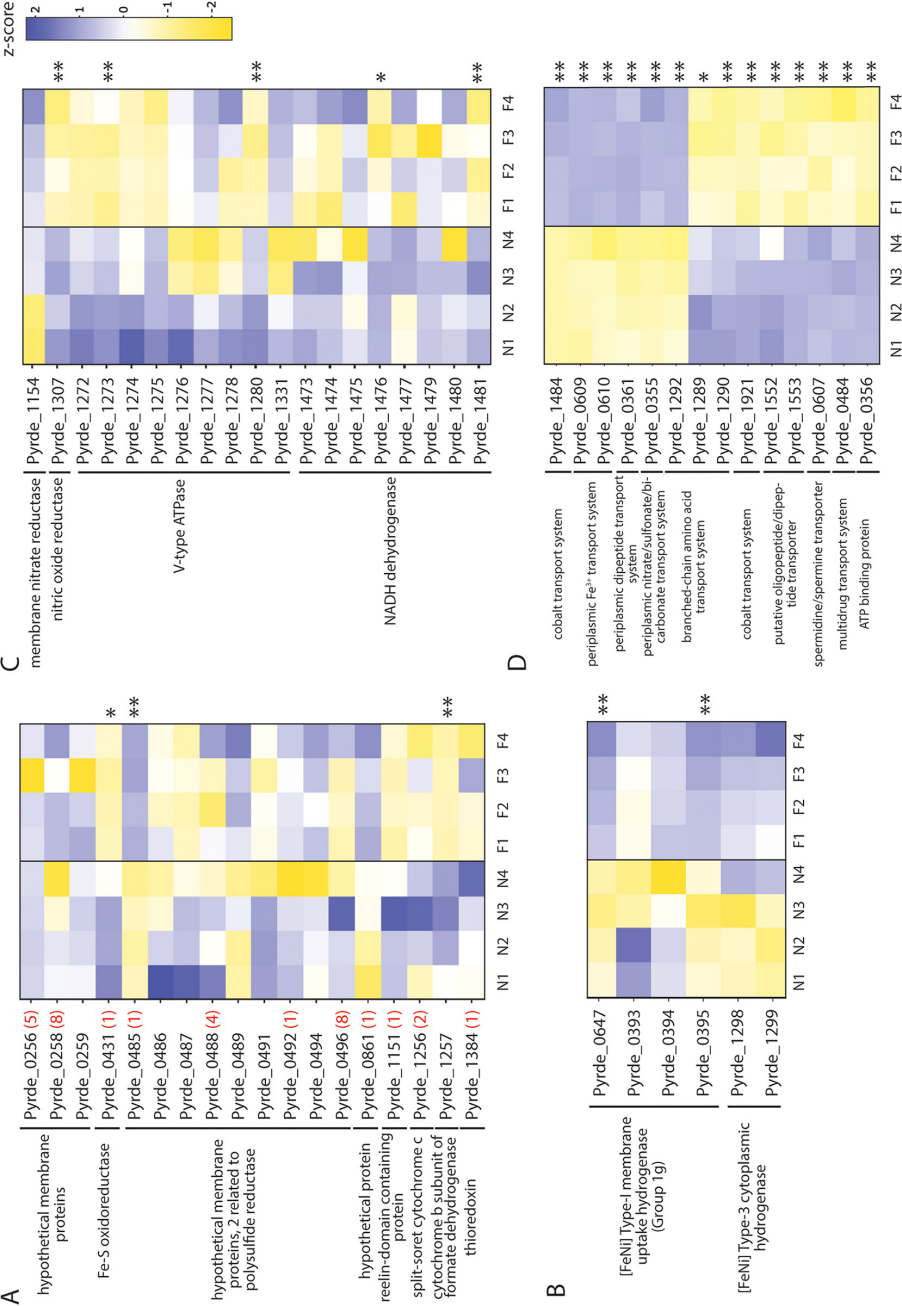
Differential analysis and heatmap of *c*-type cytochrome and associated proteins (A), membrane and cytoplasmic hydrogenase proteins (B), select redox enzymes (C), and ABC-type membrane transporters identified in nitrate- (N1 to N4) and ferrihydrite-grown (F1 to F4) cells (D). The numbers in red indicate the number of CXXCH heme motifs in that protein. ** and *, differentially produced proteins with log_2_ fold change greater than 1 or less than −1 (|log2FC| > 1) with adjusted *P* values of <0.01 and <0.05, respectively, during ferrihydrite relative to nitrate growth. A Z-score scale is used to represent higher and lower abundances compared to the mean for that protein across all samples in shades of blue and yellow, respectively.

Differences in protein abundance were examined for all *c*-type cytochrome and associated proteins, membrane and cytoplasmic hydrogenase proteins, other redox enzymes, and ABC-type membrane transporters (Fig. 4). Of the 17 predicted *c*-type cytochromes, a total of 13 *c*-type cytochromes were detected (Fig. 4A). In addition to the two *c*-type cytochrome proteins described above, a hypothetical protein containing one heme motif (Pyrde_0485) and part of a putative membrane-bound respiratory complex (Pyrde_0485-Pyrde_0496) showed greater abundance in ferrihydrite growth (Fig. 4A; see also Fig. S1). Only one *c*-type cytochrome protein, a monoheme Fe-S oxidoreductase (Pyrde_0431) showed higher abundance in nitrate-grown cells (Fig. 4A; see also Table S2). No significant differences in abundance were found for the other *c*-type cytochromes (Fig. 4A; see also Table S3). Among *c*-type cytochrome-associated proteins, a cytochrome *b* subunit of formate dehydrogenase (Pyrde_1257) was significantly more abundant in nitrate-grown cells (Fig. 4A; see also Table S2). This protein is associated with a diheme split-Soret cytochrome *c* (Pyrde_1256) that was identified in both growth conditions but was not differentially abundant (Table S3).

The *P. delaneyi* genome also encodes for a type I membrane uptake hydrogenase (group 1g) and a type 3 cytoplasmic hydrogenase. Both hydrogenases were produced in ferrihydrite- and nitrate-grown cultures, but only the catalytic subunits HcaL/HyaB (Pyrde_0647) and HcaS/HyaA (Pyrde_0395) of the membrane hydrogenase were 7- and 4-fold more abundant, respectively, in ferrihydrite-grown cells (Fig. 4B; see also Table S1), suggesting potential coupling of H_2_ oxidation and ferric iron or nitrate reduction. There were no differences in the abundances of a putative periplasmic nitrate reductase (Pyrde_1154), but a nitric oxide reductase (Pyrde_1307) was significantly more abundant in nitrate-grown cells (Fig. 4C; see also Table S2). Similarly, only two subunits of a V-type ATPase (Pyrde_1273 and 1280) and two subunits of NADH dehydrogenase (Pyrde_1476 and Pyrde_1481) were more abundant in nitrate-grown cells (Fig. 4C; see also Table S2).

There were no changes in the abundance of flagellin proteins. Analysis of the *P. delaneyi* genome with FlaFind (v.1.2) (http://signalfind.org/flafind.html) (14, 15) and PilFind (v.1.0) (http://signalfind.org/pilfind.html) (16) identified 26 archaeal class III signal peptides and 4 bacterial type IV pilin-like signal peptides and their prepilin peptidase cleavage sites. However, all of these were not detected, not tagged, or not significantly different for the two growth conditions. Nine of these proteins contained ≥9.8% aromatic amino acids, and of these, 4 also showed no aromatic gaps >22 amino acids. These criteria were used previously to identify putative e-pili from nonmodel organisms (17–20). None of these proteins were homologous or shared sequence identity to electroactive pili in *Geobacter* species, and only one (Pyrde_1193) showed low sequence identity (34.3% identity) with the conductive archaellum of Methanospirillum hungatei JF-1 (18). This protein showed lower aromatic density (5% aromatics and aromatic gaps as large as 58 amino acids) than is typical for electrically conductive filaments. Therefore, *P. delaneyi* does not appear to use conductive pili for extracellular electron transfer.

Proteomic analysis showed that several ABC-type transporters were more abundant during ferrihydrite- and nitrate-dependent growth (Fig. 4D; see also Table S1 and S2). There was a >14,700-fold increase in a putative membrane cobalt transport protein (Pyrde_1484) in ferrihydrite-grown cells (Fig. 4D; see also Table S1). However, a different putative membrane cobalt transport protein (Pyrde_1921) was more abundant in nitrate-grown cells (Table S2). Two proteins that are part of a periplasmic Fe^3+^ transport system (Pyrde_0609 and Pyrde_0610), likely for iron acquisition and assimilation, increased in abundance when cells were grown on ferrihydrite (Fig. 4D; see also Table S1). Additionally, a protein that is part of a periplasmic nitrate/sulfonate/bicarbonate transport system (Pyrde_0355) was higher in abundance in ferrihydrite-grown cultures, but the ATP binding protein associated with this system (Pyrde_0356) showed higher abundance in nitrate cultures (Fig. 4D). Both nitrate- and ferrihydrite-grown cells showed several specific oligopeptide and amino acid transport proteins, typically affiliated with quorum sensing.

### Enzyme activities.

Specific activities of redox, tricarboxylic acid cycle, and other enzymes were measured to confirm their presence in nitrate-grown cell extracts and are listed in Table 1. Methyl viologen- and NAD(P)^+^-dependent hydrogenases (H_2_ oxidizing), nitrate reductase, and ferredoxin:NADH oxidoreductase showed activity. Only 2-oxoglutarate oxidoreductase and succinyl-coenzyme A (CoA) synthetase showed measurable activity among the tricarboxylic acid cycle enzymes. Malic enzyme, pyruvate oxidoreductase, and AMP-forming acetyl-CoA synthetase also showed activity. Formate dehydrogenase, citrate synthase, aconitase, isocitrate dehydrogenase, fumarate reductase, fumarase, malate dehydrogenase, and ADP-forming acetyl-CoA synthetase did not show any activity despite genes annotated for them in the genome.

**TABLE 1:** Specific activities and identification in TMT experiment of redox and tricarboxylic acid cycle enzymes at 80°C using whole-cell protein extracts of *P. delaneyi* grown on nitrate

Enzyme	ORF no.	In proteome (yes or no)	Activity (nmol min^−1^ mg^−1^)
Hydrogenase, membrane type I	Pyrde_0647, Pyrde_0393-Pyrde_0402	Yes, Yes	7,960 ± 810
Hydrogenase, cytoplasmic type III	Pyrde_1299	Yes	159 ± 54
Nitrate reductase	Pyrde_1154-Pyrde_1156	Yes/Yes^*a*^^*b*^/No	798 ± 78
Formate dehydrogenase	None		0
Ferredoxin:NAD oxidoreductase	Pyrde_1807	No	535 ± 49
ATP citrate lyase	None		ND^*d*^
Citrate synthase	Pyrde_1539	No	0
Aconitase	Pyrde_0502-Pyrde_0503	No/Yes	0
Isocitrate dehydrogenase	Pyrde_1536	Yes^*a*^^*c*^	0
2-oxoglutarate oxidoreductase	Pyrde_1713-Pyrde_1714	Yes^*a*^^*b*^	277 ± 127
Succinyl-CoA synthetase	Pyrde_0793-Pyrde_0794	No	395 ± 14
Fumarate reductase	Pyrde_1917-Pyrde_1920	No/No/No/Yes^*a*^^*b*^	0
Fumarase	Pyrde_1915-Pyrde_1916	Yes^*a*^^*c*^	0
Malate dehydrogenase	None		0
Acetyl-CoA synthetase (AMP-forming)	Pyrde_0366	Yes	19 ± 4
Acetyl-CoA synthetase (ADP-forming)	Pyrde_0750, Pyrde_1729	Yes^*a*^^*c*^/Yes^*a*^^*b*^	0
Pyruvate oxidoreductase	Pyrde_0267-Pyrde_0270, Pyrde_1446-Pyrde_1449	Yes, Yes	34 ± 21
Malic enzyme	Pyrde_1925	Yes^*a*^^*b*^	171 ± 10

a Peptides were identified but TMT labeling was limited/absent.

b Two or more peptides identified in at least 50% of samples (medium-high confidence identification).

c One peptide identified in less than 50% of samples (low-confidence identification).

d ND, no data.

## DISCUSSION

Mineral-based microbial metabolism is ubiquitous in various geothermal environments (21). However, known mechanisms for iron reduction and energy generation are limited. Nearly all studies of hyperthermophilic iron reduction have focused on the following six species of archaea: *G. ahangari* (8) and *F. placidus* (9), both *Archaeoglobaceae* in the *Euryarchaeota*, and *P. islandicum* (7), *P. aerophilum* (10), *Acidianus* strain DS80 (11, 20), and *P. delaneyi* (5, 12) in the *Crenarchaeota*. They all use the Fe(III) oxide mineral ferrihydrite as a terminal electron acceptor, and all but *P. delaneyi* use the soluble iron form Fe(III) citrate. This study demonstrated that *P. delaneyi* required direct mineral contact for ferrihydrite reduction, did not appear to produce endogenous shuttles or chelators, and was unable to use the exogenous shuttles or chelators that were provided. This finding is consistent with its inability to use soluble iron for growth. Many hyperthermophilic iron reducers required direct contact for mineral reduction (7–9), suggesting that this is a widespread phenomenon in hyperthermophilic archaea at circumneutral pH.

Prior to this study, it was unknown if multiheme *c*-type cytochromes or other redox proteins were produced in *P. delaneyi* during growth on iron or nitrate. When the proteomes of *P. delaneyi* were compared, three putative membrane-bound *c*-type cytochrome proteins (Pyrde_0485, Pyrde_0784, Pyrde_0921) were more abundant in ferrihydrite-grown cells than in nitrate-grown cells. Pyrde_0784, an 8-heme *c*-type cytochrome, is part of a putative membrane-bound respiratory complex that also contains a 13-heme *c*-type cytochrome subunit for which differential abundance information was not available. While this putative cytochrome complex lacks homology with any proteins from *Pyrobaculum*, *Acidianus*, *Geoglobus*, and *Ferroglobus*, it is homologous to a putative respiratory complex of unknown function in Pyrolobus fumarii (another *Pyrodictiaceae*) that contains 11- and 6-heme *c*-type cytochrome proteins (Pyrfu_0785 to Pyrfu_0788). The *Pyrodictiaceae* are composed of autotrophs (22–24) and peptide-utilizing organotrophs (25, 26) that grow by sulfur (except *P. delaneyi* Su06^T^), nitrate, and iron reduction. Therefore, this cytochrome complex may be a common part of membrane electron transport in many *Pyrodictiaceae*.

Two putative membrane-bound molybdopterin oxidoreductase complexes (Pyrde_0919-Pyrde_0921 and Pyrde_1511-Pyrde_1513) in *P. delaneyi* were more abundant in ferrihydrite-grown cells than in nitrate-grown cells and may be the primary catalyst for Fe(III) oxide mineral reduction in this organism. The lack of formate dehydrogenase enzyme activity in the whole-cell extract (Table 1) and occurrence of these proteins in phylogenetic clades that are distinct from formate dehydrogenase (see Fig. S3 in the supplemental material) indicate that these enzymes are not formate dehydrogenases. As demonstrated in previous studies (27, 28), phylogenetic analysis of molybdopterin oxidoreductase (MoOR) protein complexes (using catalytic subunits) showed distinct clades based not only on function but also taxonomy (see Fig. S3). The catalytic subunits of the two putative membrane complexes produced in higher abundance during ferrihydrite growth (Pyrde_0921 and Pyrde_1511) were found in two clades of presently unknown or undescribed function (MoOR 1 and MoOR 2).

Among the *Pyrobaculum*, these two complexes showed sequence homology with molybdopterin oxidoreductases in *P. islandicum* (Pisl_0161-Pisl_0162 and Pisl_1994-Pisl_1995) and *P. aerophilum* (PAE2859-PAE2861 and PAE2661-PAE2662). Iron reduction in *P. islandicum* and *P. aerophilum* was proposed to occur largely independent of *c*-type cytochromes (7, 10). Differential heme staining of *P. aerophilum* proteins in one-dimensional (1-D) gels showed *c*-type cytochromes decreased from two bands in nitrate-grown cells to one band in iron-grown cells (10). Similarly, differential heme staining of *P. islandicum* proteins showed one band in thiosulfate-grown cells but no bands in iron grown cells (7). Two molybdopterin oxidoreductase protein bands in silver-stained gels, a nitrate reductase from *P. aerophilum* (PAE3611 and PAE3612) and a thiosulfate reductase from *P. islandicum* (Pisl_0266), were identified by peptide mass fingerprinting and present in nitrate- and thiosulfate-grown cells, respectively, but were absent in *Pyrobaculum* cells grown on iron (7, 10). The enzyme(s) responsible for iron reduction in *Pyrobaculum* was not identified. A lack of transcriptional regulation and a specific oxidoreductase for iron reduction was proposed when *P. aerophilum* was grown on Fe(III) citrate relative to nitrate, O_2_, As(V), or no addition of terminal electron acceptor (29). An operon encoding a molybdopterin oxidoreductase complex (PAE1263-PAE1265) was upregulated during growth on arsenate and was identified as a membrane-bound arsenic reductase (29). The data presented in Cozen et al. (29) suggested that this operon as well as genes in another molybdopterin oxidoreductase-encoding operon (PAE2859-PAE2861) were moderately or partly upregulated during growth on Fe(III). Therefore, the molybdopterin oxidoreductases in *P. islandicum* and *P. aerophilum* that showed the highest homology to the two *P. delaneyi* molybdopterin oxidoreductases associated with iron reduction, which are distinct from the nitrate reductase and thiosulfate reductase identified previously, may be involved in iron reduction in these crenarchaea.

The involvement of molybdopterin oxidoreductases in iron reduction has not been reported previously for any microbe. Active expression of molybdopterin oxidoreductase proteins was shown among iron oxidizers such as Mariprofundus ferrooxydans PV-1 (30). These proteins are part of membrane-bound alternative complex (AC) III respiratory complexes (31). A recent study suggested that these pterin-containing enzymes could serve as a metabolic signature of iron metabolism (32). This study examined many iron oxidizers but only one iron reducer, Shewanella oneidensis MR-1, which showed a signal lower than that found for iron oxidizers. Examining iron reducers, such as *P. delaneyi*, which show elevated production of molybdopterin oxidoreductase proteins when grown on ferrihydrite, could yield a more prominent metabolic signature for life detection in this regard among iron reducers. Future experiments would need to confirm this possibility.

Using the classification system of Greening et al. (33), all of the subunits for a type 1g membrane uptake hydrogenase (Pyrde_0647, Pyrde_0393-0395) and a type 3 cytoplasmic hydrogenase (Pyrde_1298-Pyrde_1299) were detected in the TMT proteomic analysis with two subunits of the membrane hydrogenase more abundant when cells were grown on iron (Fig. 4B). Benzyl viologen- and NADP^+^-linked hydrogenase activities, generally representing membrane and cytoplasmic H_2_-uptake hydrogenase activities, respectively, were measured in whole-cell extracts with benzyl viologen-dependent activity being larger than NADP^+^-dependent activity (Table 1). These data confirm that *P. delaneyi* is a hydrogenotroph, which cannot grow without the addition of H_2_ (5).

**FIG 5:**
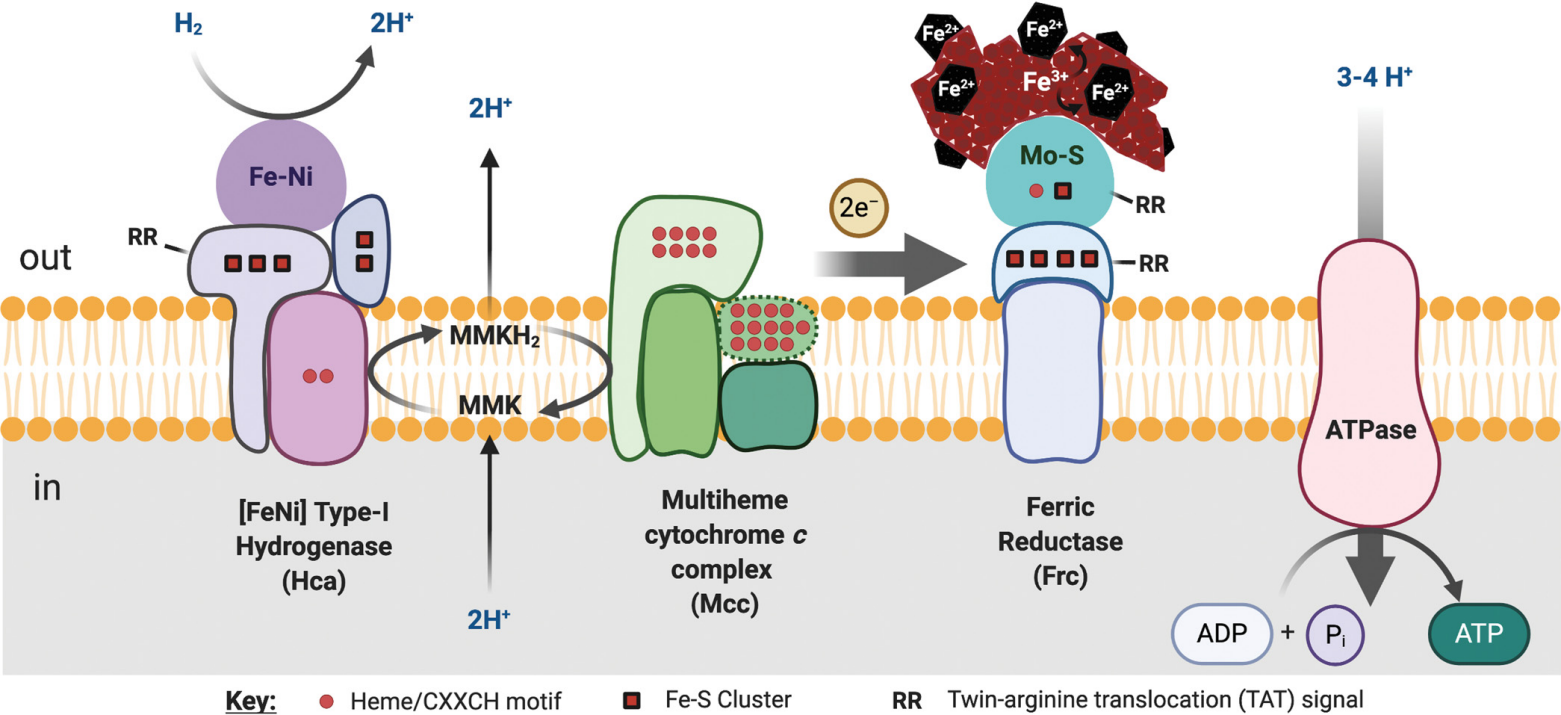
Proposed membrane electron transport chain of *P. delaneyi* grown on ferrihydrite based on proteomic analysis. Hca, [FeNi] type I membrane uptake hydrogenase (Pyrde_0647, Pyrde_0393-Pyrde_0395); Mcc, putative multiheme cytochrome *c* containing respiratory complex (Pyrde_0784-Pyrde_0787); Frc, putative molybdopterin-containing ferric reductase (Pyrde_0919-Pyrde_0921 [shown] or Pyrde_1511-Pyrde_1513); ATPase, V-type ATP synthase (Pyrde_1272-Pyrde_1280, Pyrde_1330-Pyrde_1335); MMK and MMKH_2_, oxidized and reduced methylmenaquinones (34). RR represents twin-arginine translocation (TAT) sequence, red circles depict CXXCH motifs/heme groups, red squares depict 4Fe-4S or 2Fe-2S clusters, and dashed lines are used where abundance/presence of protein is not known.

Within the *Pyrodictiaceae*, *P. fumarii* possessed methylmenaquinone (MMK) as a membrane electron carrier (34), and the key enzyme for its synthesis, menaquinone methyltransferase (35), was present in the *P. delaneyi* proteomic analysis (Pyrde_0835; see also Table S3). Furthermore, all of the subunits of a V-type ATP synthase were present in the proteomic analysis (Fig. 4C). We propose that *P. delaneyi* generates energy by oxidizing H_2_ on the outside of the membrane (based on a twin-arginine transport [TAT] signal), the hydrogenase passes the electrons to MMK and consumes two protons from the cytoplasm, and MMK passes the electrons to the membrane cytochrome complex (Mcc) identified herein that is unique to the *Pyrodictiaceae* with concomitant expulsion of two protons outside the cell (Fig. 5). The protons generated from H_2_ oxidation and the MMK drive oxidative phosphorylation through the V-type ATP synthase. When *P. delaneyi* is grown on ferrihydrite, Mcc passes the electrons to the two molybdopterin oxidoreductases (FrcI and FrcII), whose catalytic subunits face the outside of the cell based on TAT signals, which transfer the electrons to ferrihydrite through direct contact and reduce Fe^3+^ to Fe^2+^ (Fig. 5). A Nap-like membrane nitrate reductase (Pyrde_1154) and a membrane nitric oxide reductase (Pyrde_1307) were detected by TMT proteomic analysis and nitrate reductase activity was measured (Table 1), but only the latter protein was more abundant in nitrate grown cells (Fig. 4C). We propose that these enzymes are similarly fed electrons by MMK and Mcc or another cytochrome containing oxidoreductase. Genes involved in denitrification showed limited variation in expression across all growth conditions in *P. aerophilum* as well (29), suggesting that this is possibly a common phenomenon across crenarchaea.

Although *P. delaneyi* is an obligate hydrogenotroph typically grown with CO_2_, it was unclear if it fixes CO_2_. Its genome has most of the genes for the reverse tricarboxylic acid cycle except ATP citrate lyase and malate dehydrogenase, but these enzyme steps could be bypassed using citrate synthase in reverse (36, 37), AMP-forming ATP synthetase, pyruvate oxidoreductase, and malic enzyme. Detection of tricarboxylic acid cycle enzymes was weak or absent using proteomics, and only succinyl-CoA synthetase and 2-oxoglutarate oxidoreductase enzyme activities were detected among the tricarboxylic acid cycle enzymes in whole-cell extract (Table 1). AMP-forming ATP synthetase, pyruvate oxidoreductase, and malic enzyme activities were also present, and collectively these five enzymes are likely involved in biosynthesis reactions but not CO_2_ fixation. Therefore, *P. delaneyi* appears to use the 0.02% (wt vol^−1^) yeast extract present in the growth medium for biosynthesis and is likely a chemolithoheterotroph. The presence of NADP^+^-dependent hydrogenase and ferredoxin:NADH oxidoreductase activities in whole-cell extracts (Table 1) suggests that these enzymes help maintain redox balance in the cytoplasm.

In conclusion, *P. delaneyi* appears to couple H_2_ oxidation with ferrihydrite or nitrate reduction using membrane molybdopterin oxidoreductases and a unique membrane cytochrome complex. This report suggests that molybdopterin oxidoreductases may be used for Fe(III) oxide mineral reduction, which may extend to other crenarchaea, but further examination of the proteins involved is necessary for validation. The need for H_2_ flux, organic compounds, and direct contact with Fe(III) oxide minerals for growth on iron helps to constrain the environments where hyperthermophilic iron reduction can occur. Seawater ingress into hydrothermal metal sulfide deposits leads to Fe(III) oxide mineral formation in the interior pore spaces of the deposits (5). H_2_ in the hydrothermal fluid and organic compounds from nearby macrofauna and microbial biofilms or generated abiotically can provide the other compounds needed for growth. In the case of ferrihydrite, nanophase magnetite (Fe_3_O_4_) is formed as a mineral product (5, 12). A combined understanding of physiological mechanisms and mineral products involved in this metabolism may improve our ability to model and detect hyperthermophilic iron reducers in natural samples.

## MATERIALS AND METHODS

### Growth media and culture conditions.

*P. delaneyi* Su06^T^ (DSM 28599) (13) was grown in modified DSM 981 medium. The base medium was composed of the following (per liter): 19 g of NaCl, 9 g of MgCl_2_·6H_2_O, 0.3 g of CaCl_2_·2H_2_O, 0.5 g of KCl, 0.14 g of KH_2_PO_4_, 0.05 g of NaBr, 0.02 g of SrCl_2_·6H_2_O, 0.15 g of MgSO_4_·7H_2_O, 0.1 g of (NH_4_)_2_SO_4_, 1 g of NaHCO_3_, 0.2 g yeast extract (Difco, vitamin fortified), 10 ml of DSM medium 141 trace element solution, and 10 ml of DSM medium 141 vitamin solution. Either 50 mmol liter^−1^ of ferrihydrite or 1 g liter^−1^ of potassium nitrate was used as terminal electron acceptors for all protein experiments. A total of 100 mmol liter^−1^ of ferrihydrite was used in all iron barrier experiments. Ferrihydrite was synthesized as described previously (38). The medium was pH balanced to 6.80 ± 0.05 (room temperature), and 0.025% cysteine-HCl was added prior to inoculation as the reducing agent. For the protein experiments, cultures were incubated in 2-liter Chemglass bottles containing 1.2 liter of growth medium, sealed with butyl rubber stoppers, and degassed and flushed with H_2_:CO_2_ (80%:20% ratio). Bottles were inoculated using a logarithmic growth phase culture that had been grown and transferred at least three times on the electron acceptor for that experiment. After inoculation, bottles were overpressurized with 2 atm of H_2_:CO_2_ (80%:20% ratio) and incubated with stirring at 90°C in a forced-air incubator.

### Iron barrier experiments.

Ferrihydrite was separated from *P. delaneyi* using dialysis tubing—Spectra/Por 2 (molecular weight cutoff [MWCO], 12 to 14 kDa)—as previously described (10). This prevented direct contact between cells and minerals but allowed diffusion of low-molecular-weight shuttles or chelators. *P. delaneyi* was grown in 50 ml of growth medium contained in 160-ml serum bottles using either ferrihydrite in dialysis tubing, free ferrihydrite suspensions, or free ferrihydrite plus empty dialysis tubing. The third condition was included to ensure that the dialysis tubing or clips did not inhibit growth. These conditions were also tested with the separate addition of either an artificial electron shuttle, 50 μM anthraquinone-2,6-disulfonate (AQDS); a metal chelator, 4 mM nitrilotriacetic acid (NTA); or 75% (vol vol^−1^) of cell-free spent supernatant to fresh medium. The cell free spent supernatant was harvested from late logarithmic growth-phase cultures grown on ferrihydrite and filtered through 0.2-μm pore size filters. Cell, Fe^2+^, and total iron concentrations were determined using epifluorescence microscopy and ferrozine assays, respectively, at various time points throughout each experiment as described previously (10, 12).

### Proteomic analysis.

Total protein was extracted from 8 cell pellets, 4 from each growth condition (ferrihydrite versus nitrate). Each pellet was obtained from 1.2 liters of cells grown until late logarithmic growth phase. Cell pellets were collected by centrifugation at 10,000 × *g* for 1 h at 4°C and washed two times with 50 mM Tris-HCl, pH 8.0 buffer. For cells grown with ferrihydrite, prior to centrifugation, 250 ml of TPE buffer (100 mM Tris-HCl, 10 mM EDTA, 300 mM KH_2_PO_4_, pH 7.0) and 1 liter of oxalate solution (226 mM ammonium oxalate, 167 mM oxalic acid) were added to dissolve the iron minerals and dissociate cells from the mineral particles. To ensure thorough mixing without compromising protein integrity, this step was performed on ice for 20 min. The harvested pellet was stored at −20°C until further use. Cell lysis, protein extraction, reduction, alkylation, acetone precipitation, and in-solution protein digestion were performed using the Pierce mass spec sample prep kit for cultured cells (Thermo Scientific, Rockford, IL, USA). Prior to beginning protocols as described by the manufacturer, cell pellets were thawed and disrupted on ice by sonication. Cell lysis was confirmed using phase-contrast light microscopy, and this suspension was processed through the kit. Extracted proteins were quantified using the DC protein assay kit (Bio-Rad, Hercules, CA, USA). Bovine serum albumin was used as a protein standard.

Differential protein abundances were determined quantitatively by labeling equal amounts of digested peptides (50 μg) using TMT10plex isobaric label reagents (Thermo Scientific, Rockford, IL, USA). This labeling kit can be used to label up to 10 different peptide samples for identification and quantification using mass spectrometry. Eight isobaric tags (126, 127N, 127C, 128N, 128C, 129N, 129C, 130N) were used to label four biological replicates per growth condition, and each condition was technically replicated with a different isobaric label. Labeled peptide samples associated with each technical replicate were combined in equal amounts, resulting in two samples containing eight isobarically labeled biological samples (4 for ferrihydrite and 4 for nitrate). The combined samples were analyzed two times (instrumental technical replicates) using a Thermo Easy-nLC 1000 nanoscale liquid chromatography (nanoLC) system at the University of Massachusetts Mass Spectrometry Center. Briefly, 5 μl of sample was loaded onto a trap column and desalted with 15 μl buffer A (buffer A, 0.1% formic acid in water; buffer B, 0.1% formic acid in acetonitrile). Peptides were then eluted through a 50-cm nanocolumn (PepMap RSLC, 500 mm by 75 μm; Thermo) over 180 min using a gradient from 5% B to 45% B at a flow rate of 300 nl min^−1^ into the mass spectrometer. Eluted peptides were detected with an Orbitrap Fusion (Thermo Electron). MS1 spectra were acquired at 120,000 resolution over a range of 375 to 1,500 *m/z* with a 2-s cycle time. Data-dependent tandem mass spectrometry (MS/MS) spectra for peptide identification were acquired in the linear ion trap using an isolation width of 1.2 *m/z* and collision-induced dissociation (CID) with normalized collision energy (NCE) of 35%. TMT quantitation was achieved by synchronous precursor selection (SPS) of the top 5 precursors (MS1 charge state, +2; isolation width, 1.3 *m/z*) or top 10 precursors (MS1 charge state, +3; isolation width; 0.7 *m/z* or MS1 charge state, +4 to +6; isolation width, 0.5 *m/z*) and subsequent MS3 using high-energy collisional dissociation (HCD) at 65% NCE with Orbitrap detection at 50,000 resolution.

RAW instrument files were processed using Proteome Discoverer v.2.3 software. Instrumental technical replicates were merged and searched with the SEQUEST HT search engine with the *P. delaneyi* Su06^T^ proteome (GenBank accession number CP013011). The search was configured with static modifications for the TMT reagents (+229.163 Da) on lysines and N termini, carbamidomethyl (+57.021 Da) on cysteines, dynamic modifications for oxidation of methionine residues (+15.995 Da), precursor mass tolerance of 10 ppm, fragment mass tolerance of 0.6 Da, and trypsin cleavage with a maximum of 2 missed cleavage sites. A reversed sequence decoy strategy was used to control peptide false discovery, and identifications were validated by Percolator software. Only peptides with *q* values of ≤0.05 were used, and at least one unique peptide was required for reporting identified proteins. Reporter ions were corrected for isotopic impurities (specific to the lot of label reagents provided by Thermo Scientific) and quantified with a coisolation threshold of 75, and synchronous precursor selection (SPS) mass matches a threshold of 65%. Protein abundances were quantified by summing the reporter ion intensities across all matching peptide spectrum matches (PSMs). The abundance values were normalized to the total peptide amount for each reporter ion channel to account for equal protein loading. Normalized reporter ion intensities from Proteome Discoverer v.2.3 were used to identify differentially produced proteins within the software as well as using edgeR in the Bioconductor software framework and the debrowser package in R (v.3.6.1 [http://www.r-project.org]) (39, 40). Low intensity proteins were filtered to exclude proteins for which maximum intensities for each protein across all conditions were less than 10. Trimmed mean of M values (TMM) normalization was performed using the edgeR package. edgeR uses an empirical Bayes dispersion estimation, fits a negative binomial generalized log-linear model, and performs protein-wise statistical tests. Proteins were reported as differentially produced if |log2FC| > 1, where FC is fold change and the adjusted *P* value is <0.01.

Protein samples were also separated by electrophoresis and visualized using both silver and heme staining. Equal amounts of protein from whole-cell extracts of ferrihydrite- and nitrate-grown cells were boiled for 5 min and separated by electrophoresis in tris-tricine buffered, 8% polyacrylamide gels. Total proteins were silver stained for imaging using the Pierce silver stain for mass spectrometry kit (Thermo Scientific, Rockford, IL, USA). Protein samples were also separated in gels omitting reducing agents and boiling to identify proteins containing *c*-type heme groups. Heme groups were identified by measuring peroxidase activity using H_2_O_2_ and 3,3′,5,5′-tetramethylbenzidine (TMBZ) as previously described (41). The 160-kDa and 140-kDa heme bands from nitrate and ferrihydrite, respectively, were excised from the gel, destained using a sodium sulfite solution as previously described (41), and digested overnight with trypsin using the in-gel tryptic digestion kit (Thermo Scientific, Rockford, IL, USA). The digested peptide fragments were analyzed using a Thermo Orbitrap Fusion Tribrid mass spectrometer equipped with ultrahigh-performance liquid chromatography (UHPLC) coupled with electrospray ionization and tandem mass spectrometry (nano-LC-ESI-MS*^n^*) at the University of Massachusetts Mass Spectrometry Center. MS data were analyzed using the Proteome Discoverer v.2.3.

### Enzyme activities.

All enzyme assays were performed using whole-cell extracts, which were transferred and manipulated in an anoxic chamber and using degassed and N_2_ flushed sample buffers containing 2 mM sodium dithionite (DT). A 1.2-liter nitrate-grown late-logarithmic-growth-phase culture was harvested by centrifugation as described above, and the pellet was resuspended in anoxic 50 mM Tris-HCl (pH 8.0) buffer containing DT. The pellet was placed in an anoxic vial and frozen at −20°C until further use. The cell suspension was thawed on ice, and DNase I was added at a final concentration of 0.0002% (wt vol^−1^). The cells were then sonicated on ice, and lysis verified by phase-contrast microscopy. Protein concentrations were determined spectrophotometrically using the DC protein assay kit (Bio-Rad, Hercules, CA, USA). Bovine serum albumin was used as a protein standard. The whole-cell extract was anoxically aliquoted into several vials and stored at −20°C.

All enzyme activities were measured at 80°C using glass or quartz cuvettes in a BioMate 6 UV-visible (UV-Vis) spectrophotometer (Thermo Fisher Scientific, Waltham, MA, USA) with a Thermo Haake DC10 circulating water bath (Thermo Electron, Waltham, MA, USA) attached to the cuvette holder or by discontinuous assays. The following anaerobic enzyme activities were measured in containers flushed with N_2_: benzyl viologen- and NADP^+^-dependent hydrogenase (42, 43), nitrate reductase (44), fumarase (45), fumarate reductase (46), 2-oxoglutarate oxidoreductase (47), aconitase (48), pyruvate oxidoreductase (47), formate dehydrogenase (49), and ferredoxin:NADH oxidoreductase (42). The following aerobic enzyme activities were measured: malate dehydrogenase (50), succinyl-CoA synthetase (51), isocitrate dehydrogenase (50), citrate synthase (52), ADP- and AMP-forming acetyl-CoA synthetase (53, 54), and malic enzyme (55). A detailed description of the enzyme assays is provided in the supplemental material.

### Data availability.

The mass spectrometry proteomics data have been deposited to the ProteomeXchange Consortium (http://proteomecentral.proteomexchange.org) via the PRIDE partner repository (56) with the data set identifier PXD020757 and 10.6019/PXD020757. Data used for differential protein analyses are additionally included in Tables S1 to S4 in the supplemental material.

## Supplementary Material

Supplemental file 1

Supplemental file 2
