# Supplementary material for: Microbe-Mineral Interaction and Novel Proteins for Iron Oxide Mineral Reduction in the Hyperthermophilic Crenarchaeon Pyrodictium delaneyi
Source: Appl Environ Microbiol. 2021 Feb 26;87(6):e02330-20. doi: 10.1128/AEM.02330-20 (PMC8105010; doi:10.1128/AEM.02330-20)
Supplement: Supplemental file 1 [file AEM.02330-20-s0001.pdf]

Supplementary Information

Microbe-Mineral Interaction and Novel Proteins for Iron Oxide Mineral Reduction in the Hyperthermophilic Crenarchaeon *Pyrodictium delaneyi*

Srishti Kashyap and James F. Holden

This file contains:

3 Supplementary Figures
Detailed Description of Enzyme Assays

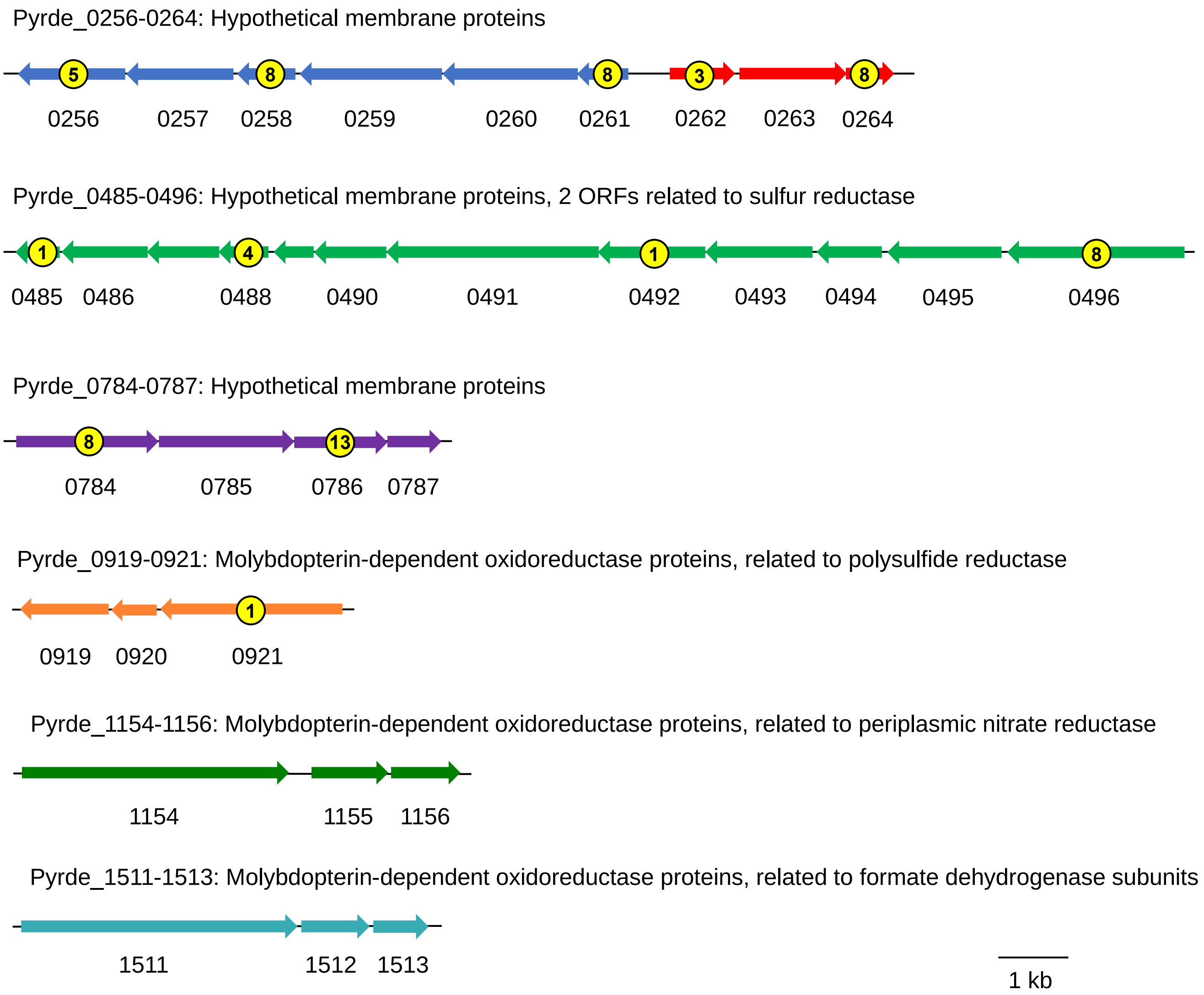

Fig. S1. *P. delaneyi* operon map showing genes in potential operons that encode for putative membrane-bound respiratory complexes. Genes for putative *c*-type cytochrome-containing proteins are highlighted with yellow circles. The numbers in the yellow circles indicate the number of CXXCH motifs in each protein encoded by the gene. The numbers below the arrows are gene identifiers.

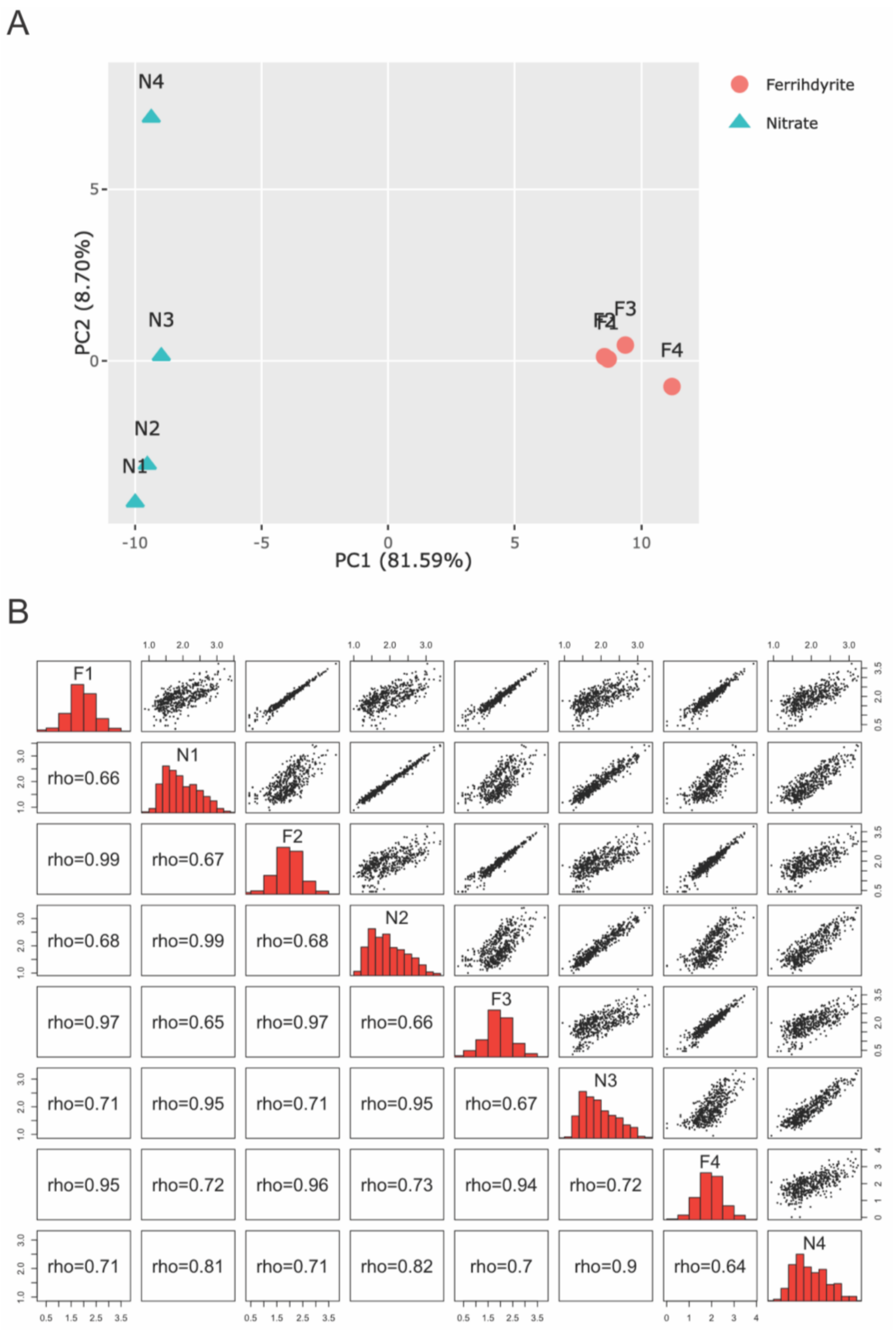

Fig. S2. Principle component analysis (PCA) (A) and correlation plots (B) for replicates of ferrihydrite (F1-4) and nitrate (N1-4) grown cells used for proteomic analysis.

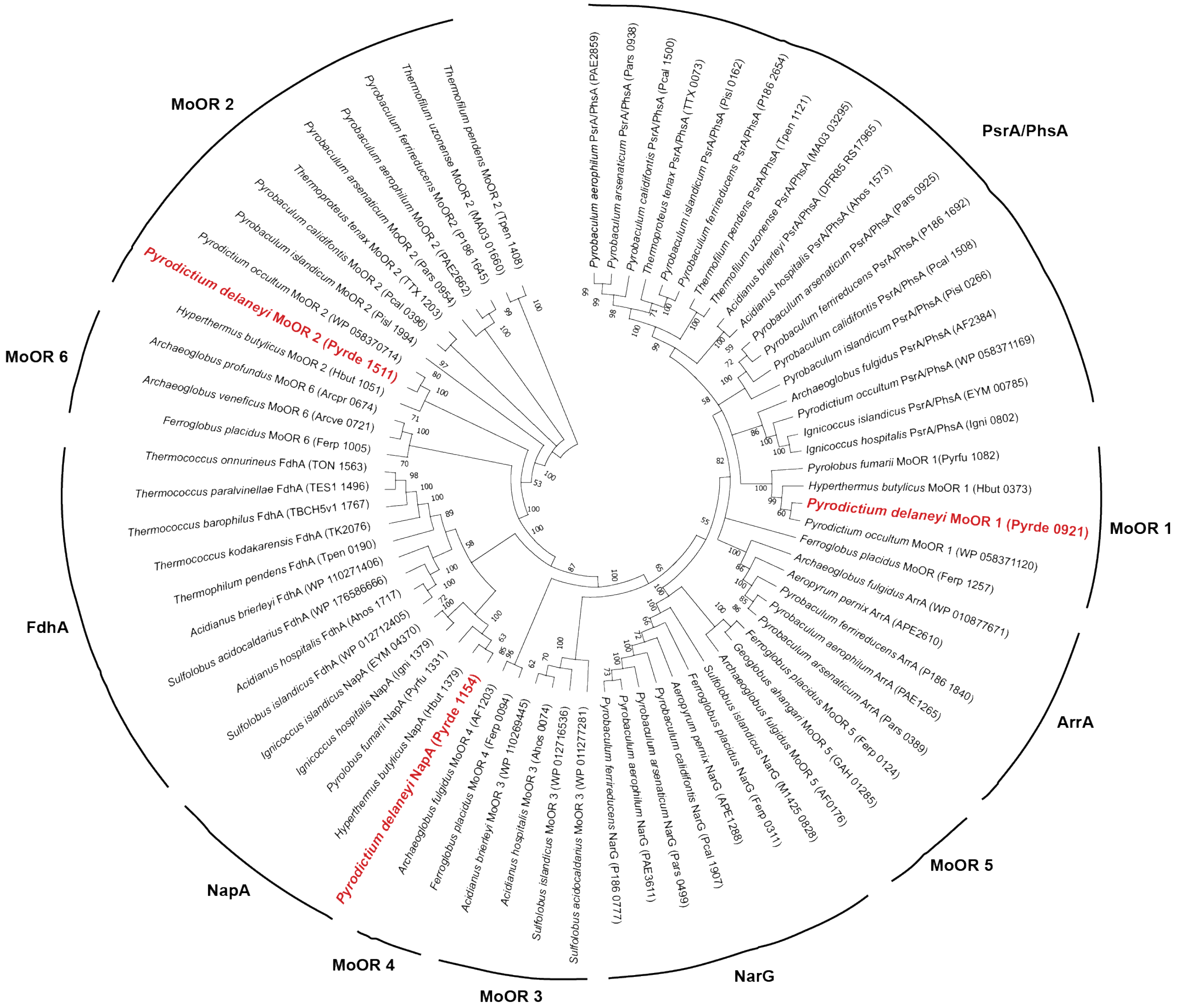

Fig. S3. Phylogenetic tree of catalytic subunits in molybdopterin oxidoreductases (Mo-ORs) across the Archaea. The three protein sequences in *Pyrodictium delaneyi* are shown in red and bold. The unrooted tree was constructed using the maximum likelihood method and bootstrap values inferred from 500 replicates. The number at each node represents the percentage of replicate trees in which the associated proteins clustered together in the bootstrap analysis. The analysis involved 72 amino acid sequences. There were a total of 2216 positions in the final dataset. Evolutionary analyses were conducted in MEGA X. ArrA, arsenate reductase; FdhA, formate dehydrogenase; Nap, periplasmic nitrate reductase; NarG, respiratory nitrate reductase; PsrA/PhsA, polysulfide/polythionate reductase.

Enzyme Activity Assays

All enzyme activity assays were performed using whole cell extracts, which were transferred and manipulated in an anoxic chamber and using degassed and N_2 flushed sample buffers containing 2 mM sodium dithionite (DT). A 1.2-liter nitrate grown late-logarithmic growth phase culture was harvested by centrifugation, and the pellet was resuspended in anoxic 50 mM Tris-HCl (pH 8.0) buffer containing DT. The pellet was placed in an anoxic vial and frozen at -20°C until further use. The cell suspension was thawed on ice and DNase I was added at a final concentration of 0.0002% (wt vol^{-1}). The cells were then sonicated on ice, and lysis verified by phase-contrast microscopy. Protein concentrations were determined spectrophotometrically using the DC Protein Assay kit (Bio-Rad, Hercules, CA, USA). Bovine serum albumin was used as a protein standard. This whole-cell extract was anoxically aliquoted into several vials and stored at -20°C.

All enzyme activities were measured at 80°C using glass or quartz cuvettes in a BioMate 6 UV-VIS spectrophotometer (Thermo Fisher Scientific, Waltham, MA, USA) with a ThermoHaake DC10 circulating water bath (Thermo Electron, Waltham, MA, USA) attached to the cuvette holder or by discontinuous assays in 50 mM *N*-(2-hydroxyethyl) piperazine-*N'*-3-propanesulfonic acid (EPPS) buffer (pH 8.4) unless otherwise stated.

The following enzyme activity assays were performed anoxically in degassed and N_2 flushed rubber-stoppered cuvettes. Hydrogenase activity was determined by measuring reduction of either 1 mM benzyl viologen (BV) at 600 nm (ε = 7400 M^{-1} cm^{-1}) or 1 mM $NADP^+$ at 340 nm (ε = 6220 M^{-1} cm^{-1}) in cuvettes that had been flushed with H_2 (1, 2). 2-oxoglutarate oxidoreductase (OGOR) and pyruvate oxidoreductase (POR) activities were determined by measuring the reduction of 1 mM methyl viologen (MV) at 578 nm (ε = 9,700 M^{-1} cm^{-1}) using 5 mM 2-oxoglutarate and 10 mM pyruvate, respectively, as the substrates (3). The assay mixtures also contained 2.5 mM $MgCl_2$, 0.4 mM thiamine pyrophosphate (TPP), and 0.1 mM CoASH. Fumarate reductase (FR) activity was determined by measuring the oxidation of 1 mM BV reduced with 25 µM DT at 578 nm (ε = 8,650 M^{-1} cm^{-1}) using 1 mM fumarate as the substrate (4). Aconitase activity was determined by measuring the change in absorbance of *cis*-aconitate at 240 nm (ε = 3,500 M^{-1} cm^{-1}) following the addition of sample (5). The assay mixture contained 1 mM isocitrate as substrate in quartz cuvettes. Nitrate reductase (NR) activity was determined by measuring the oxidation of 0.3 mM BV that had been reduced with 0.24 mM DT at 600 nm (ε = 7,400 M^{-1} cm^{-1}) using 10 mM sodium nitrate as the substrate (6). The assay was performed in 100 mM potassium phosphate buffer (pH 7.0). Fumarase activity was determined by measuring the change of absorbance of fumarate at 250 nm (ε = 1,479 M^{-1} cm^{-1}) following the

addition of sample (7). The assay was performed in 100 mM potassium phosphate buffer (pH 7.2) and the mixture also contained 50 mM malate. Formate dehydrogenase (FDH) activity was determined by measuring the reduction of 5 mM BV at 600 nm (ε = 7,400 M^{-1} cm^{-1}) using using 10 mM sodium formate as the substrate (8). Ferredoxin:NAD^+ oxidoreductase (FNOR) activity was determined by measuring reduction of 1mM BV at 600 nm (ε = 7,400 M^{-1} cm^{-1}) using 0.3 mM NADH as the substrate (1). A trace amount of 100 mM DT (10 µl) was also added. The assay was run in 50 mM CAPS (pH 10.3).

The following enzyme activity assays were performed under oxic conditions. Isocitrate dehydrogenase (IDH) activity was determined by measuring the reduction of 0.4 mM $NADP^+$ at 340 nm (ε = 6,220 M^{-1} cm^{-1}) using 0.1 mM isocitrate as the substrate (9). The assay mixture also contained 5 mM $MgCl_2$. Decarboxylating malic enzyme (MAE) activity was determined in 50 mM Tris-HCl (pH 8.0) buffer by measuring the reduction of 0.05 mM $NADP^+$ at 340 nm (ε = 6,220 M^{-1} cm^{-1}) using 1 mM malate as the substrate (10). The assay mixture also contained 0.1 mM $MnCl_2$. Citrate synthase (CS) activity was determined by measuring the release of CoASH using 0.25 mM 5,5'-dithiobis-2-nitrobenzoic acid (DTNB) as the CoASH-detecting agent at 412 nm (ε = 13,600 M^{-1} cm^{-1}) and 0.2 mM oxaloacetate as the substrate (11). The assay mixture also contained 0.2 mM acetyl-CoA. ADP- and AMP-forming acetyl-CoA synthetase (ACS) activities were determined by measuring the release of CoASH using 0.25 mM DTNB at 412 nm (ε = 13600 M^{-1} cm^{-1}) (12, 13). The ADP-dependent reaction was performed in 100 mM MOPS buffer (pH 7.0), and the assay mixture contained 5 mM $MgCl_2$, 2 mM ADP, 5 mM KH_2PO_4 and 0.2 mM acetyl-CoA to start the reaction (12). The AMP-dependent reaction was measured in 100 mM MES buffer (pH 6.5) and the assay mixture contained 0.5 mM AMP, 1 mM pyrophosphate, and 0.1 mM acetyl-CoA to start the reaction (13). Malate dehydrogenase (MDH) activity was determined by measuring the oxidation of 0.4 mM NADH at 340 nm (ε = 6,200 M^{-1} cm^{-1}) using 0.4 mM oxaloacetate as the substrate (9). Succinyl-CoA synthetase (SCS) activity was determined by measuring phosphate formation using 5 mM succinate as substrate (14). The reaction was initiated by adding 1 mM ATP and quenched after 2, 4, and 6 min with 0.1 ml of 6 N H_2SO_4. The amount of phosphate produced was measured spectrophotometrically as described previously (15). The assay mixture also contained 10 mM $MgCl_2$ and 0.2 M KCl and 0.1 mM CoASH.

References

1. Ma K, Adams MWW. 2001. Hydrogenases I and II from *Pyrococcus furiosus*. Method Enzymol 331:208–216.

2. van Haaster DJ, Silva PJ, Hagedoorn P-L, Jongejan JA, Hagen WR. 2008. Reinvestigation of the steady-state kinetics and physiological function of the soluble NiFe-Hydrogenase I of *Pyrococcus furiosus*. J Bacteriol 190:1584–1587.

3. Schut GJ, Menon AL, Adams MWW. 2001. 2-keto acid oxidoreductases from *Pyrococcus furiosus* and *Thermococcus litoralis*. Method Enzymol 331:144–158.

4. Beh M, Strauss G, Huber R, Stetter K-O, Fuchs G. 1993. Enzymes of the reductive citric acid cycle in the autotrophic eubacterium *Aquifex pyrophilus* and in the archaebacterium *Thermoproteus neutrophilus*. Arch Microbiol 160:306–311.

5. Uhrigshardt H, Walden M, John H, Anemüller S. 2001. Purification and characterization of the first archaeal aconitase from the thermoacidophilic *Sulfolobus acidocaldarius*. Eur J Biochem 268:1760–1771.

6. Afshar S, Kim C, Monbouquette HG, Schröder I. 1998. Effect of tungstate on nitrate reduction by the hyperthermophilic archaeon *Pyrobaculum aerophilum*. Appl Environ Microbiol 64:3004–3008.

7. Mizobata T, Fujioka T, Yamasaki F, Hidaka M, Nagai J, Kawata Y. 1998. Purification and characterization of a thermostable class II fumarase from*Thermus thermophilus*. Arch Biochem Biophys 355:49–55.

8. Ma K, Loessner H, Heider J, Johnson MK, Adams MW. 1995. Effects of elemental sulfur on the metabolism of the deep-sea hyperthermophilic archaeon *Thermococcus* strain ES-1: characterization of a sulfur-regulated, non-heme iron alcohol dehydrogenase. J Bacteriol 177:4748–4756.

9. Steen IH, Madern D, Karlström M, Lien T, Ladenstein R, Birkeland NK. 2001. Comparison of isocitrate dehydrogenase from three hyperthermophiles reveals differences in thermostability, cofactor specificity, oligomeric state, and phylogenetic affiliation. J Biol Chem 276:43,924–43,931.

10. Bartolucci S, Rella R, Guagliardi A, Raia CA, Gambacorta A, De Rosa M, Rossi M. 1987. Malic enzyme from archaebacterium *Sulfolobus solfataricus.* Purification, structure, and kinetic properties. J Biol Chem 262:7725–7731.

11. Danson MJ, Hough DW. 2001. Citrate synthase from hyperthermophilic archaea. Method Enzymol 331:3–12.

12. Bräsen C, Schönheit P. 2004. Unusual ADP-forming acetyl-coenzyme A synthetases from the mesophilic halophilic euryarchaeon *Haloarcula marismortui* and from the hyperthermophilic crenarchaeon *Pyrobaculum aerophilum*. Arch Microbiol 182:277–287.

13. Bräsen C, Urbanke C, Schönheit P. 2005. A novel octameric AMP-forming acetyl-CoA synthetase from the hyperthermophilic crenarchaeon *Pyrobaculum aerophilum*. FEBS Lett 579:477–482.

14. Hutchins AM, Holden JF, Adams MWW. 2001. Phosphoenolpyruvate synthetase from the hyperthermophilic archaeon *Pyrococcus furiosus*. J Bacteriol 183:709–715.

15. Hasegawa H, Parniak M, Kaufman S. 1982. Determination of the phosphate content of purified proteins. Anal Biochem 120:360–364.
